# Clinically relevant safety issues associated with St. John's wort product labels

**DOI:** 10.1186/1472-6882-8-42

**Published:** 2008-07-17

**Authors:** Kevin A Clauson, Marile L Santamarina, Jennifer C Rutledge

**Affiliations:** 1 Nova Southeastern University, College of Pharmacy – West Palm Beach, 3970 RCA Boulevard, Suite 7006A, Palm Beach Gardens, FL 33410, USA; 2 Lloyd L. Gregory School of Pharmacy, Palm Beach Atlantic University, West Palm Beach, FL 33416, USA

## Abstract

**Background:**

St. John's wort (SJW), used to treat depression, is popular in the USA, Canada, and parts of Europe. However, there are documented interactions between SJW and prescription medications including warfarin, cyclosporine, indinavir, and oral contraceptives. One source of information about these safety considerations is the product label. The aim of this study was to evaluate the clinically relevant safety information included on labeling in a nationally representative sample of SJW products from the USA.

**Methods:**

Eight clinically relevant safety issues were identified: drug interactions (SJW-HIV medications, SJW-immunosupressants, SJW-oral contraceptives, and SJW-warfarin), contraindications (bipolar disorder), therapeutic duplication (antidepressants), and general considerations (phototoxicity and advice to consult a healthcare professional (HCP)). A list of SJW products was identified to assess their labels. Percentages and totals were used to present findings.

**Results:**

Of the seventy-four products evaluated, no product label provided information for all 8 evaluation criteria. Three products (4.1%) provided information on 7 of the 8 criteria. Four products provided no safety information whatsoever. Percentage of products with label information was: SJW-HIV (8.1%), SJW-immunosupressants (5.4%), SJW-OCPs (8.1%), SJW-warfarin (5.4%), bipolar (1.4%), antidepressants (23.0%), phototoxicity (51.4%), and consult HCP (87.8%). Other safety-related information on labels included warnings about pregnancy (74.3%), lactation (64.9%), discontinue if adverse reaction (23.0%), and not for use in patients under 18 years old (13.5%). The average number of *a priori *safety issues included on a product label was 1.91 (range 0–8) for 23.9% completeness.

**Conclusion:**

The vast majority of SJW products fail to adequately address clinically relevant safety issues on their labeling. A few products do provide an acceptable amount of information on clinically relevant safety issues which could enhance the quality of counseling by HCPs and health store clerks. HCPs and consumers may benefit if the FDA re-examined labeling requirements for dietary supplements.

## Background

St. John's wort (*Hypericum perforatum*), used most commonly for depression, is one of the top selling and best researched herbs used in the USA [1-3]. It is also popular in Canada and many countries in Europe – including Germany, where it is used more frequently than selective serotonin reuptake inhibitors (SSRIs) [4,5]. In addition to St. John's wort's (SJW) primary use in depression, there is increasing interest in research for it in related mental conditions such as obsessive compulsive disorders and social phobia [6]. While conflicting evidence exists about its efficacy, SJW has demonstrated its most positive results in mild-to-moderate depression with some success seen in treating major depression [6,7]. Hypericin and hyperforin are the components of SJW to which its antidepressant activity is most commonly ascribed. However, most clinical trials have used SJW extracts standardized to 0.3% hypericin content with a dose of 300 mg three times a day, although other dosing regimens have been used [8]. SJW is believed to exert its antidepressant action by activating the serotonergic, noradrenergic, and dopaminergic systems and by activating gamma-aminobutyric acid (GABA) and glutamic acid receptors in the brain [9,10]. Since SJW is a pharmacologically active agent, it also is responsible for adverse reactions and has been associated with multiple potentially serious and clinically relevant drug interactions. Concurrent use of SJW has been implicated in decreasing plasma levels of cyclosporine A resulting in organ rejection in transplant patients [11,12]. In HIV patients, it has decreased plasma levels of indinavir resulting in failure of antiretroviral therapy [13]. Women taking SJW and oral contraceptives could experience a decrease in efficacy resulting in an increased number of breakthrough bleeding events and unplanned pregnancies [14]. Concomitant use of SJW with warfarin has been associated with decreased anticoagulation properties resulting in unstable or decreased International Normalized Ratio (INR) values and is reportedly responsible for decreased plasma levels of digoxin [15]. Use of SJW is also associated with the induction of mania or hypomania in patients suffering with bipolar disorder [16]. It has been postulated that the drug interactions observed with the co-administration of SJW and prescription medication such as those described above are mediated by different mechanisms. Most of these interactions are consistent with the induction of cytochrome P450 3A4 enzyme. Further studies indicate that other likely mechanisms of SJW drug-herb interactions exist, including enhanced expression and drug efflux activity of P-glycoprotein/MDR1 [12,17] and activation of pregnane X [18,19]. Additionally, use of SJW has been associated with reversible photosensitivity causing erythematous skin lesions after sun exposure [10].

Despite a fairly well-defined literature and an increase in educational initiatives, there is still a considerable lack of knowledge about clinically relevant safety issues associated with herbs such as St. John's wort among healthcare professionals (HCPs) [20-23] and employees at health food stores [24,25] where these products are often purchased. Moreover, many consumers still mistakenly perceive that the use of herbal supplements, also called "natural remedies", carry little to no risk [26,27]. In addition to general evaluations of herbal knowledge among HCPs, knowledge specific to SJW has also been assessed. One study of physicians revealed that those surveyed were unaware of interactions involving SJW including: SJW-warfarin (56%), SJW-digoxin (68%), and SJW-OCP (68%) [28]. Similarly, a study of community pharmacists found that 48% were unaware of the interaction between SJW and cyclosporine, and 32% had not heard of the SJW-OCP interaction [4].

Since 1994, dietary supplements (i.e. herbs, nutraceuticals, botanicals) have been regulated under the Dietary Supplement Health and Education Act (DSHEA) in the USA [29]. Under DSHEA, manufacturers are not required to provide data demonstrating safety and efficacy of a dietary product as long as it was present in the marketplace prior to the introduction of the legislation. As such, the Food and Drug Administration (FDA) can only intervene if a dietary supplement is proven unsafe after it has already reached consumers. Even then, the onus is on the FDA to demonstrate that the product is unsafe, rather than on the manufacturer to produce evidence of its safety.

Dietary supplement manufacturers in the USA also include or omit safety information on product labels at their own discretion. The lack of clinically pertinent information displayed on the label of natural health products has recently been addressed in countries such as Canada where 71% of the population reports using natural products [4]. The new Canadian regulatory measures now require more specific information to be included on labels for natural health products including potential drug interactions and adverse effects [30]. The benefits of including clinically relevant safety information on dietary supplement product labels was demonstrated in a recent study by Sarino et al [14]. Inclusion of drug interaction information on product labels was associated with a lower incidence of inappropriate advice dispensed about SJW products by both pharmacists and health store clerks. Based on these findings, more comprehensive safety warnings on the labeling of SJW products may be positively associated with improved quality of counseling by pharmacists and advice from health store clerks. This would help improve patient safety by increasing the likelihood of an intervention before an adverse event occurs. The purpose of this study was to evaluate clinically relevant safety information included on labeling in a nationally representative sample of SJW products in the USA.

## Methods

### Evaluation Criteria

An exhaustive search was conducted of the primary literature using secondary databases including MEDLINE, EMBASE, IPA, MANTIS, and AltHealthWatch for evidence of safety issues involving SJW. Tertiary resources such as AltmedDex, Facts & Comparisons Review of Natural Products, Lexi-Natural, Natural Medicines Comprehensive Database, and Natural Standard were also reviewed. Safety issues were classified as one of four categories: 1) SJW – drug interactions, 2) SJW – contraindications, 3) SJW – therapeutic duplication, and 4) SJW – general. Consideration was given to those safety issues which were documented based on level of evidence, likelihood of exposure, and/or those that were theoretical or mechanistic in nature but presented the potential for serious risk. SJW label evaluation criteria were finalized and are listed in Additional file 1. Additional safety information from the label was recorded for all products.

### Compilation of products

Sources for compiling the list of SJW products included: Whole Foods Source Book, Natural Standard, Natural Medicines Comprehensive Database, ConsumerLab.com, and Amazon.com. The top 20 retail pharmacy companies, by pharmacy sales, were also searched for store brand SJW products. SJW products were identified and then compiled into a master list on an Excel spreadsheet. Field headings of the spreadsheet included: manufacturer, product, dosage, constituents, uniform resource locator (URL), and miscellaneous notes along with those headings from Additional file 1.

Only those SJW products produced as a single-ingredient entity (i.e. no combination products with other herbs) and commercially available in the USA were eligible for inclusion. Oral formulations including tablets, capsules, and caplets were included. SJW products available as creams, liquids, oils, sprays, teas, and tinctures were excluded. Homeopathic preparations of SJW were also excluded. Manufacturers with multiple single-ingredient SJW products were limited to one representative product. If multiple products in one line existed, the SJW product containing a dose of 300 mg was selected as the default product. If no 300 mg version was available, the next closest dose was selected as that manufacturer's representative. Products originating from the U.K. and Canada that were priced in pounds or Canadian dollars, or labeled according to requirements in their respective country, were also excluded.

### Evaluation process

Guidelines were established with one author performing evaluations and another rechecking a subset for quality assurance. For example, credit was given in either case if the label recommended consulting a "healthcare professional" or named a specific type of practitioner (e.g. physician, pharmacist, etc.). This was not the case for related but non-specific label warnings (e.g. in drug interactions category, a generic warning about taking with "prescription drugs" did not earn credit for the specific warnings for drugs or classes that were being assessed). Credit was given for a drug interaction if either the drug class or specific drug was named (e.g. if the label mentioned warfarin or Coumadin^® ^or 'blood thinners' it was given credit for the SJW-warfarin interaction). Only external product labeling was considered for this evaluation.

## Results

### St. John's wort products

Seventy-four products were identified as available for purchase online, by phone, or at local retailers that met criteria. No product provided all 8 label safety indicators. However, the most complete labeling information was observed for three (4.1%) of the products. Each of those three provided information on 7 of the 8 safety criteria – including all of the drug-herb interactions. Four products provided no safety information whatsoever. The percentages of products with label information including the respective safety issues were as follows: SJW-HIV medications (8.1%), SJW-immunosupressants (5.4%), SJW-oral contraceptives (OCPs) (8.1%), SJW-warfarin (5.4%), bipolar (1.4%), antidepressants (23.0%), phototoxicity (51.4%), and consult HCP (87.8%). Full results of the clinically relevant safety information from each product label are provided in Additional file 1. Other safety-related information (recorded in the notes field of the Excel spreadsheet) that appeared most frequently included warnings about pregnancy (74.3%), lactation (64.9%), to discontinue if an adverse reaction occurred (23.0%), and that it was not for use in patients less than 18 years old (13.5%). Additional safety-related information noted on SJW labels were warnings about: discontinuing two weeks prior to surgery, allergies to ragweed/daisy-like flowers, avoiding tyramine-containing foods, and use with severe kidney disease, liver disease, or Parkinson's. Specific side effects were also mentioned including: anxiety, dizziness, drowsiness, gastrointestinal, insomnia, restlessness, tiredness, and vivid dreams.

The average number of safety issues included on a product label was 1.91 (range 0–8) for 23.9% completeness of the eight criteria determined *a priori*. Additionally, of the four safety issues catalogued after the evaluation, the average label score was 1.76 (44.0%). A combination of the eight pre-specified and four additional safety issues on labels had an average score of 3.66 (range 0–12) for 30.5% completeness.

## Discussion

It is a promising finding that the vast majority of SJW products included some type of prompt for consumers to consult a HCP in conjunction with taking the herb. However, the effectiveness of this prompt is contingent upon the HCP being knowledgeable about the requisite safety issues. Unfortunately, studies thus far have indicated that this necessary knowledge is not universally possessed [4,28]. Additionally, specificity of label safety information, rather than a blanket warning, has been more closely tied to making a positive impact on the quality of advice dispensed about these products [14]. This provision of specific, clinically relevant safety information on the label is where the SJW products performed the most poorly in this study. Since no warning within the drug interaction category even reached 10% inclusion on all SJW labels, the potential for negative impact on patient safety remains. If HCPs and health store clerks are uninformed about these interactions and have even disavowed knowledge of their existence in some cases [14], how many preventable adverse events are occurring where incomplete product safety labeling is a contributing factor? This could be particularly problematic as the initial point of contact for a consumer interested in purchasing a dietary supplement is often a pharmacist or health store clerk. Additionally, health food store personnel have even been identified as a source of information ranking higher than physicians, dietitians, and other HCPs when consumers consider using herbal products [31].

Complicating matters further is that SJW and its components have been studied in treating HIV due to its antiretroviral properties *in vitro *[32]. While this is likely a minor issue as the compound was administered intravenously and not found efficacious, the possibility exists HIV patients will take SJW to treat their HIV/AIDS which could compromise their conventional cocktails/therapy. This possibility is heightened by the fact that some SJW products are labeled with this type of indication (e.g. "has been skillfully crafted to promote antiviral/antibacterial properties..." is one such statement found on the study product labels). Alarmingly, information and recommendations of SJW to treat HIV persist on the Internet in both scientific and non-scientific sources that could send mixed messages to consumers and health care professionals [33,34].

While the majority of products performed below expectations, the labeling of three products did stand out positively. SJW products by Douglas Labs, Nature Made, and New Chapter all included each of the four drug interaction alerts and 7 of the 8 safety issues overall. Since this is an industry largely driven by self-regulation, it is of interest why these three outliers contained the breadth of safety issues that the other products did not include on their labels. Since Douglas Labs specifically targets HCPs rather than consumers with their product lines, they may be more in-tune with the clinically oriented needs of physicians and pharmacists. They also consult with their scientific advisory board and insurance carrier to help develop label recommendations. Nature Made has partnered with the American Botanical Council (ABC) in order to determine which safety information to include and uses an accordion-style label to help display the relevant information. Finally, New Chapter relies on their in-house scientific counsel and review process along with direction from the American Herbal Products Association (AHPA) to generate a more complete label. All three approaches have been successful in that they have all produced a label far more complete than the industry average in addressing clinically relevant safety issues.

The possibility exists that selectively targeting products such as these top performers for recommendations or inclusion in product inventories at pharmacies, health stores and physician groups could result in better informed consumers, employees, and HCPs and ultimately improves the quality of advice dispensed. This, of course, would be a short-term solution as different market forces could act to interfere with this line of action. The absence of relevant safety warnings on SJW product labels resembles results from the assessment of aspirin-related safety warnings on white willow bark product labels, which demonstrated a similar dearth of safety information [35]. In that case, the lack of aspirin-warnings on a product label likely contributed to a negative clinical outcome and an emergency department admission [36]. The poor label performances seen in this study should herald potential dangers to the Food and Drug Administration (FDA), HCPs, manufacturers, and consumers. The self-regulating nature of the dietary supplement industry under the auspices of DSHEA, and minimal FDA involvement, has resulted in a very broad range of safety warnings included on the product labels of herbal products. More unified and comprehensive regulatory measures for dietary supplement labels could yield safer use of these supplements and result in more complete information available to consumers, HCPs, and health store personnel.

### Limitations

The sample of products used in this study was limited to those commercially available in the USA, thus results cannot be extrapolated to products in other countries. Additionally, not every interaction, side effect, and safety-related issue that has been documented or proposed regarding SJW was assessed in this study. The inclusion or exclusion of certain safety issues could have affected the scores of different SJW products. However, given that three products had labels that were so much more complete than the rest of the products, any changes in scores would likely have been minor and not have substantially affected the study results. Finally, as this was not an interventional study, it is uncertain exactly how the use of products with a high percentage of safety issues on the labels would translate into actual patient benefit and adverse event prevention.

## Conclusion

The vast majority of SJW products fail to adequately address clinically relevant safety issues on their labeling. A small number of products do provide an acceptable amount of information on drug-herb interactions and other safety issues which could enhance the quality of advice and counseling by HCPs and health store clerks. Both HCPs and consumers may ultimately realize the greatest benefit if the FDA were to re-examine labeling requirements of dietary supplements.

## Competing interests

KAC received financial support for acquisition of products, conducting the evaluation and dissemination of results, including the article processing fee, from Pharmavite LLC., which includes the Nature Made brand.

## Authors' contributions

KAC conceptualized the overall project, designed and directed collection of the data, and coordinated the preparation of this manuscript. MLS assisted in conceptualizing the project and participated in the data analysis and conceptualization of this manuscript. JCR assisted in conceptualizing the project and assisted in data analysis. All authors critically edited drafts of this manuscript and approved the submitted manuscript.

## Pre-publication history

The pre-publication history for this paper can be accessed here:



## Supplementary Material

Additional file 1 Table 1. SJW label data for categories of safety issues. Click here for file
