# Supplementary material for: Clinically relevant safety issues associated with St. John's wort product labels
Source: BMC Complement Altern Med. 2008 Jul 17;8:42. doi: 10.1186/1472-6882-8-42 (PMC2483264; doi:10.1186/1472-6882-8-42)
Supplement: Additional file 1 — Table 1. SJW label data for categories of safety issues. [file 1472-6882-8-42-S1.doc]

# Tables

**Table 1. SJW label data for categories of safety issues**

| **Manufacturer** | **HIV medications** | **Immunosuppressants** | **Oral contraceptives** | **Warfarin** | **Bipolar disorder** | **Antidepressants** | **Phototoxicity** | **Consult HCP** | **Discontinue if ADR** | **Avoid in <18 years old** | **Lactation/Nursing** | **Pregnancy** |
| --- | --- | --- | --- | --- | --- | --- | --- | --- | --- | --- | --- | --- |
| **21st Century** |  |  |  |  |  |  |  | ⬤ |  |  |  |  |
| **A to Z Naturals** |  |  |  |  |  |  | ⬤ | ⬤ |  |  | ⬤ | ⬤ |
| **Aboca USA, Inc.** |  |  |  |  |  |  | ⬤ | ⬤ |  |  | ⬤ | ⬤ |
| **ActiveHerb Technology** | ⬤ |  |  |  |  | ⬤ |  | ⬤ | ⬤ |  |  |  |
| **Bluebonnet** |  |  |  |  |  |  |  |  |  |  |  |  |
| **CVS** |  |  |  |  |  |  |  | ⬤ | ⬤ |  | ⬤ | ⬤ |
| **Daily Manufacturing, Inc.** |  |  |  |  |  |  |  |  |  |  |  |  |
| **David Edelberg, M.D.** |  |  |  |  |  |  | ⬤ | ⬤ |  |  | ⬤ | ⬤ |
| **DaVinci Laboratories** |  |  |  |  |  | ⬤ | ⬤ | ⬤ |  |  | ⬤ | ⬤ |
| **Douglas Labs (formerly AMNI)** | ⬤ | ⬤ | ⬤ | ⬤ |  | ⬤ | ⬤ | ⬤ |  |  | ⬤ | ⬤ |
| **Dr. Christopher's Original Formulas** |  |  |  |  |  | ⬤ |  | ⬤ |  |  |  |  |
| **Duane Reed** |  |  |  |  |  |  |  | ⬤ | ⬤ | ⬤ | ⬤ | ⬤ |
| **Flora, Inc.** |  |  |  |  |  |  | ⬤ | ⬤ |  |  | ⬤ | ⬤ |
| **Food Science** |  |  |  |  |  | ⬤ | ⬤ | ⬤ |  |  | ⬤ | ⬤ |
| **Gaia Herbs** |  |  |  |  |  |  |  | ⬤ |  |  | ⬤ | ⬤ |
| **Garry Null & Associates (GNA)** |  |  | ⬤ |  |  |  |  | ⬤ |  |  |  |  |
| **GNC** |  |  |  |  |  |  | ⬤ | ⬤ |  |  | ⬤ | ⬤ |
| **Good'n Natural** |  |  |  |  |  |  |  | ⬤ | ⬤ | ⬤ | ⬤ | ⬤ |
| **Herbal Medi Care** |  |  |  |  |  |  |  | ⬤ | ⬤ |  |  | ⬤ |
| **Herbs of Light** |  |  |  |  |  |  |  | ⬤ |  |  |  | ⬤ |
| **Indiana Botanic Gardens, Inc.** |  |  |  |  |  |  |  | ⬤ |  |  | ⬤ | ⬤ |
| **Karuna** |  |  |  |  |  |  | ⬤ | ⬤ |  |  | ⬤ | ⬤ |
| **Kordial Nutrients** |  |  |  |  |  |  |  | ⬤ |  |  |  |  |
| **Kroeger Herbs** |  |  |  |  |  |  |  | ⬤ |  |  |  | ⬤ |
| **Lichtwer Pharma US** | ⬤ | ⬤ |  | ⬤ |  |  | ⬤ | ⬤ |  | ⬤ | ⬤ | ⬤ |
| **Life Extension** | ⬤ |  | ⬤ |  |  |  | ⬤ | ⬤ |  |  | ⬤ | ⬤ |
| **Longs** |  |  |  |  |  |  | ⬤ | ⬤ |  |  | ⬤ | ⬤ |
| **Metabolic Response Modifiers (MRM)** |  |  |  |  |  |  |  | ⬤ |  |  |  |  |
| **Natrol** |  |  |  |  |  | ⬤ | ⬤ | ⬤ | ⬤ |  | ⬤ | ⬤ |
| **Natural Factors** |  |  |  |  |  |  | ⬤ | ⬤ |  |  | ⬤ | ⬤ |
| **Nature Made** | ⬤ | ⬤ | ⬤ | ⬤ | ⬤ |  | ⬤ | ⬤ | ⬤ |  | ⬤ | ⬤ |
| **Nature's Answer** |  |  |  |  |  |  | ⬤ | ⬤ |  |  | ⬤ | ⬤ |
| **Nature's Bounty** |  |  |  |  |  |  |  | ⬤ | ⬤ | ⬤ | ⬤ | ⬤ |
| **Nature's Herbs** |  |  |  |  |  |  | ⬤ |  |  |  |  | ⬤ |
| **Nature's Plus** |  |  |  |  |  |  |  | ⬤ |  |  | ⬤ | ⬤ |
| **Nature's Sunshine** |  |  |  |  |  |  | ⬤ | ⬤ |  |  | ⬤ | ⬤ |
| **Nature's Way** |  |  |  |  |  |  | ⬤ | ⬤ |  |  | ⬤ | ⬤ |
| **New Chapter Inc** | ⬤ | ⬤ | ⬤ | ⬤ |  | ⬤ | ⬤ | ⬤ |  | ⬤ | ⬤ | ⬤ |
| **NOW Foods** |  |  |  |  |  | ⬤ | ⬤ | ⬤ |  |  |  |  |
| **Nutraceutical Corporation** |  |  |  |  |  | ⬤ | ⬤ | ⬤ |  |  | ⬤ | ⬤ |
| **Nutraceutical Sciences Institute** |  |  |  |  |  |  |  | ⬤ |  |  | ⬤ | ⬤ |
| **NutraSanus LLC** |  |  |  |  |  |  | ⬤ | ⬤ | ⬤ |  |  |  |
| **Nutrition World** |  |  |  |  |  |  | ⬤ | ⬤ | ⬤ |  |  |  |
| **Olympian Labs** |  |  |  |  |  |  | ⬤ | ⬤ |  |  | ⬤ | ⬤ |
| **Only Natural** |  |  |  |  |  |  | ⬤ | ⬤ |  |  |  | ⬤ |
| **Pacific Biologic** |  |  |  |  |  |  |  |  |  |  |  |  |
| **Paradise Herbs** |  |  |  |  |  |  |  | ⬤ |  |  | ⬤ | ⬤ |
| **Pharmacist's Ultimate Health** |  |  |  |  |  |  |  | ⬤ |  |  | ⬤ | ⬤ |
| **Planetary Herbals** |  |  |  |  |  |  |  | ⬤ |  |  | ⬤ | ⬤ |
| **Progressive Labs** |  |  |  |  |  |  |  | ⬤ |  |  |  |  |
| **ProHealth, Inc.** |  |  |  |  |  |  | ⬤ | ⬤ |  |  |  |  |
| **Pronatura** |  |  |  |  |  |  | ⬤ |  |  |  |  |  |
| **Puritan's Pride** |  |  |  |  |  |  |  | ⬤ | ⬤ | ⬤ | ⬤ | ⬤ |
| **Radiance** |  |  |  |  |  |  |  | ⬤ | ⬤ | ⬤ | ⬤ | ⬤ |
| **R-U-Ved, Inc** |  |  |  |  |  | ⬤ | ⬤ | ⬤ |  |  | ⬤ | ⬤ |
| **Sarken Nutrition Corporation** |  |  |  |  |  | ⬤ | ⬤ | ⬤ |  |  | ⬤ | ⬤ |
| **Sci-Fit** |  |  |  |  |  | ⬤ | ⬤ | ⬤ |  |  | ⬤ | ⬤ |
| **Solaray** |  |  |  |  |  | ⬤ | ⬤ | ⬤ |  |  | ⬤ | ⬤ |
| **Solgar Vitamin and Herb** |  |  |  |  |  |  | ⬤ | ⬤ |  |  | ⬤ | ⬤ |
| **Source Naturals** |  |  |  |  |  |  |  | ⬤ |  |  | ⬤ | ⬤ |
| **Spring Valley** |  |  |  |  |  |  | ⬤ | ⬤ | ⬤ |  |  |  |
| **Starwest** |  |  |  |  |  | ⬤ |  |  |  |  |  |  |
| **Sundown** |  |  |  |  |  |  |  | ⬤ | ⬤ | ⬤ | ⬤ | ⬤ |
| **Swanson Health Products** |  |  | ⬤ |  |  |  | ⬤ | ⬤ | ⬤ |  | ⬤ | ⬤ |
| **Target** |  |  |  |  |  |  |  | ⬤ | ⬤ | ⬤ | ⬤ | ⬤ |
| **Thompson** |  |  |  |  |  | ⬤ | ⬤ | ⬤ |  |  | ⬤ | ⬤ |
| **Thorne Research** |  |  |  |  |  |  |  | ⬤ |  |  |  | ⬤ |
| **Vital Nutrients** |  |  |  |  |  |  |  | ⬤ |  |  |  | ⬤ |
| **Vitamin Power** |  |  |  |  |  |  |  |  |  |  |  |  |
| **Vitamin Research Products** |  |  |  |  |  |  | ⬤ |  |  |  | ⬤ | ⬤ |
| **Vitamin World** |  |  |  |  |  |  |  | ⬤ | ⬤ | ⬤ | ⬤ | ⬤ |
| **Walgreens** |  |  |  |  |  | ⬤ |  |  |  |  | ⬤ | ⬤ |
| **Whole Foods** |  |  |  |  |  |  |  | ⬤ |  |  | ⬤ | ⬤ |
| **Whole Health Products** |  |  |  |  |  | ⬤ | ⬤ | ⬤ |  |  |  |  |
